# Evaluation of pulse wave velocity and central systolic blood pressure in children and adolescents with chronic kidney disease

**DOI:** 10.31744/einstein_journal/2022AO6758

**Published:** 2022-04-28

**Authors:** Ana Paula Brecheret, Ana Lucia Cardoso Santos Abreu, Renata Lopes, Francisco Antônio Helfenstein Fonseca, Dirceu Solé, Maria Cristina de Andrade

**Affiliations:** 1 Universidade Federal de São Paulo São Paulo SP Brazil Universidade Federal de São Paulo, São Paulo, SP, Brazil.; 2 Escola Paulista de Medicina Universidade Federal de São Paulo São Paulo SP Brazil Escola Paulista de Medicina, Universidade Federal de São Paulo, São Paulo, SP, Brazil.

**Keywords:** Pulse wave analysis, Arterial pressure, Renal insufficiency, chronic, Cardiovascular diseases, Child, Adolescent

## Abstract

**Objective:**

Investigate pulse wave velocity and central systolic blood pressure among pediatric population with chronic kidney disease.

**Methods:**

In this cross-sectional study, 57 patients (61.4% male) aged 6.2 to 17.5 years old, 44 with nondialysis chronic kidney disease and 13 on chronic dialysis, were included in the analysis. The pulse wave velocity and the central systolic blood pressure were measured with an oscillometric device with an inbuilt ARC Solver^Ⓡ^ algorithm and were compared with previously established percentiles.

**Results:**

The prevalence of elevated pulse wave velocity was 21.1% (95%Cl: 11.4-33.9) and elevated central systolic blood pressure was 28.1% (95%CI: 17.0-41.5). According to the generalized linear model, there was a higher risk of elevated pulse wave velocity in patients undergoing chronic dialysis treatment than nondialysis chronic kidney disease patients (adjPR=4.24, 95%CI: 1.97-9.13, p=<0.001). Hypertensive patients (stage 2) had a higher risk of elevated pulse wave velocity than normotensive ones (adjPR=2.70, 95%CI: 1.05-6.95, p=0.040), as did patients younger than 12 years than the older patients (adjPR=2.95, 95%CI: 1.05-8.40, p=0.041). Hypertensive patients had a higher risk of elevated central systolic blood pressure than normotensives (adjPR=3.29, 95%Cl: 1.36-7.94), as did patients undergoing chronic dialysis treatment when comparing to nondialysis chronic kidney disease patients (adjPR=2.08, 95%Cl: 1.07-4.02).

**Conclusion:**

Younger age, dialysis, and hypertension in children are independently associated with higher pulse wave velocity. Hypertension and dialysis are independently associated with higher central systolic blood pressure.

## INTRODUCTION

Chronic kidney disease (CKD), which is characterized by injury and progressive loss of renal function, is an important public health problem in Brazil^(1)^ and worldwide.^(2)^ In the pediatric age group, CKD is rare, with a prevalence between 18 and 100 cases per million, though it has been increasing in recent decades.^(3)^ In these patients, mortality is up to 30 times higher than that in the general pediatric population, and cardiovascular disease (CVD) is the most common cause.^(4,5)^ Clinical and epidemiological studies show that although cardiovascular complications manifest especially in more advanced stages of the disease, and such changes begin early in patients with CKD.^(6,7)^

Pediatric patients with CKD have a high prevalence of traditional (*e.g*., hypertension and dyslipidemia) and nontraditional risk factors for CVD (*e.g*., inflammation, anemia, abnormalities in calcium and phosphorus metabolism).^(7,8)^ Early markers of heart disease, such as hypertrophy and dysfunction of the left ventricle, and early markers of atherosclerosis, such as increased carotid intima-media thickness and arterial stiffening, are frequent findings in adults with CKD, especially in patients undergoing dialysis treatment.^(9)^

Pulse wave velocity (PWV) is a well-established predictor of cardiovascular events in adults^(10)^ and has recently been used in pediatric patients to assess the risk of CVD.^(11)^ Central systolic blood pressure (cSBP) reflects arterial changes more accurately than peripheral blood pressure and has predictive value for cardiovascular events.^(11)^ In recent years, PWV and cSBP have been widely studied and become accepted as a simple, noninvasive, reliable, and reproducible method for determining arterial stiffness.^(10)^ In pediatric CKD, data on arterial stiffening are still scarce, so further studies in this population are necessary.

Pulse wave velocity and cSBP were measured in pediatric patients with CKD, and the associations of clinical, anthropometric, and laboratory parameters with PWV and cSBP were assessed.

## OBJECTIVE

Investigate pulse wave velocity and central systolic blood pressure among pediatric population with chronic kidney disease.

## METHODS

### Population studied

This was an observational, cross-sectional study that included 57 patients with CKD, who were followed up at the pediatric nephrology outpatient clinic of the *Escola Paulista de Medicina da Universidade Federal de São Paulo* (EPM/UNIFESP) in São Paulo. The convenience sample consisted of all patients followed up at the outpatient clinic who met the inclusion and exclusion criteria. A total of 62 patients were selected, of whom three refused to participate and two did not perform the exams, making up 57 patients in the study. The inclusion criteria were age between 6 and 18 years old, creatinine clearance <90mL/min/1.73m^2^ associated with kidney injury*, i.e*., stage 2, 3a, 3b, 4, or 5 CKD according to the kidney disease improving global outcomes (KDIGO) 2012.^(12)^ The exclusion criteria were age younger than 6 or older than 18 years old and neoplastic, infectious, or inflammatory diseases. Data were collected between May 2015 and March 2016. The study was approved by the Research Ethics Committee of UNIFESP (2130/11). The informed consent form and the consent form were obtained from all study participants and guardians.

### Pulse wave velocity and central systolic pressure

Pulse wave velocity and cSBP were measured with the automated Oscillometric Apparatus Mobil -O- Graph 24 hours PWA monitor (I.E.M., Stolberg, Germany), which uses the ARC Solver^Ⓡ^ algorithm. The device used was previously validated by direct measurements and/or tonometry, with good reproducibility in previous studies.^(13,14)^ The patients remained in the dorsal decubitus position and rested for 10 minutes before the beginning of the examination. Two measurements were taken at a 5-minute interval, and the arithmetic mean between these two measurements was used as the final measurement for analysis.

For the classification of PWV and cSBP, the percentile tables for height and sex proposed by Elmenhorst were used, which were prepared for children and adolescents using the same device.^(13)^ Values of PWV and cSBP above the 95^th^ percentiles were classified as elevated.

### Variables

Chronic kidney disease was classified according to KDIGO 2012,^(12)^ and glomerular filtration rate (GFR) was estimated using the Schwartz equation.^(15)^ The etiology of the baseline disease, the duration of CKD, the type of treatment, and the time of dialysis were collected from the medical records of the service. Blood pressure (BP) was assessed by an oscillometric measurement using a digital device (Dixtal^®^, SP, Brazil) in the same week as the PWV and cSBP measurements. The patient remained at rest for 3 to 5 minutes, and a cuff adequate to the circumference of the arm (width and length) was used at the midpoint between the olecranon and the acromion. The BP measurement was confirmed by auscultatory measurement and was classified according to the Guideline for Screening and Management of High Blood Pressure in Children and Adolescents 2017.^(16)^

The patients were evaluated by a single nutritionist using an electronic scale (Filizola^®^, SP, Brazil) and physicians using a vertical wall-mounted stadiometer. Weight and height measurements were used to calculate the body mass index for age (BMI/A) z-score and height for age (H/A) z-score, according to the World Health Organization’s (WHO) reference standard.^(17)^

Blood samples were collected after a 12-hour fast 1 to 8 days after the measurement of PWV and cSBP. The samples were analyzed in the central laboratory of the *Hospital São Paulo* EPM/UNIFESP.

The laboratory references used for data analysis were as follows: uric acid (2.4-5.7mg/dL), total calcium (8.6-10.2mg/dL), ionic calcium (1.20-1.37mmol/L), hemoglobin (12-15.5g/dL), alkaline phosphatase (6 years old <269U/L, 7-12 years old <300U/L, girls 13-17 years old <187U/L and boys 13-17 years old <390U/L), phosphorus (2.5-4.5mg/dL), albumin (4 days to 14 years old 3.8-5.4g/dL and 14 to 18 years old 3.2-4.5g/dL), magnesium (1.8-2.5mg/dL), C-reactive protein (up to 1.00mg/L), 25OH-vitamin D (>30ng/dL), and sodium bicarbonate (23-27mmol/L).

For total cholesterol, LDL, HDL, and triglycerides (TG), the I Guidelines for the Prevention of Atherosclerosis in Childhood and Adolescence were used as reference.^(18)^ The Brazilian Guidelines for Clinical Practice for Mineral and Bone Disorders in Chronic Kidney Disease of Children were used for the classification of parathyroid hormone (PTH) values.^(19)^

### Statistical analysis

The prevalence of children and adolescents with CKD who had changes in PWV and cSBP, and their respective 95% confidence intervals (95%CI) were estimated. The qualitative variables were written as number (n) and percentage (%). Pearson’s correlation test was used to assess the association with PWV and cSBP. To estimate the strengths of the associations between PWV alteration or cSBP alteration and risk factors, the prevalence ratio (PR) and its 95% CI were calculated using the generalized linear model (GLM) with a binomial distribution and the log link function.^(20)^ In this case, the increase in PWV or in cSBP were considered the dependent variable, and the various exposure factors were considered independent variables. The variables that showed a p value of 20% or lower in the univariate analysis were selected to compose the multivariate model. It was decided to calculate the PR instead of calculating the odds ratio because the events evaluated (elevated PWV and elevated cSBP) were frequent (≥20%).

All analyses were performed in Stata version 14.2 for Windows. Results with p<0.05 were considered significant.

## RESULTS

The age of the studied patients (n=57) ranged from 6.2 to 17.5 years old, with median of 11.9 years old, and 61.4% of them were male. A total of 44 (77.2%) patients underwent conservative treatment, 5 (8.8%) patients underwent peritoneal dialysis, and 8 (14%) hemodialysis. Table 1 shows the main characteristics of the patients studied. Of the 17 patients on CKD stage 5, eight were on hemodialysis, five on peritoneal dialysis, and four under conservative treatment.

**Table 1:** Characteristics and cardiovascular measures of the 57 patients with stage 2 to 5 chronic kidney disease

Patient characteristic	Stage 2	Stage 3a	Stage 3b	Stage 4	Stage 5
Number	10	8	12	10	17
Boys, %	80.0	75.0	58.3	60.0	47.1
Age^*^, years	12.2 (2.6)	12.7 (4.6)	11.5 (3.3)	10.5 (3.6)	11.8 (3.4)
Time of diagnosis of CKD^†^ years	2.3 (1.5-7.0)	7.7 (3.2-10.5)	6.0 (3.6-9.2)	6.6 (4.8-12.0)	3.5 (1.6-7.0)
Uropathy/Glomerulopathy/other, %	20.0/0.0/80.0	62.5/12.5/25.0	83.3/0.0/16.7	60.0/10.0/30.0	47.1/29.4/23.5
H/A z-score*	-0.36 (1.54)	-1.94 (2.75)	-0.73 (1.66)	-1.83 (1.48)	-1.59 (2.02)
BMI/A z-score*	-0.81 (3.08)	0.18 (1.58)	-0.31 (1.15)	-0.13 (1.15)	-1.31 (1.37)
BP normal/elevated/stage hypertension 1/stage 2 hypertension, %	60.0/10.0/30.0/0.0	62.5/12.5/25.0/0.0	58.3/8.3/16.7/16.7	30.0/0.0/70.0/0.0	47.1/11.8/17.6/23.5
PWV^*^, m/s	4.44 (0.52)	4.59 (0.39)	4.42 (0.61)	4.27 (0.37)	4.64 (0.49)
Elevated PWV, %	10.0	12.5	8.3	20.0	41.2
cSBP^*^, mmHg	96.7 (16.0)	102.2 (12.0)	98.3 (18.7)	93.2 (14.4)	104.1 (15.5)
Elevated cSBP, %	10.0	50.0	16.7	20.0	41.2

* data are represented as mean (standard deviation); ^†^ data are represented as median (p25%-p75%).

H/A z-score: score and height for age; BMI/A: body mass index for age; BP: blood pressure; PWV: pulse wave velocity; cSBP: central systolic blood pressure; CKD: chronic kidney disease.

The prevalence of elevated PWV and cSBP found in the study population were 21.1% (95%CI: 11.4-33.9) and 28.1% (95%CI: 17.0-41.5), respectively.

According to table 2, sex, duration of disease, stages of CKD H/A z-score and BMI/A z-score showed no association with elevated PWV, but age, etiology, type of treatment (conservative or dialysis), time on dialysis, and stage were significantly associated with elevated PWV. Patients with glomerulopathies had a higher prevalence of elevated PWV (57.1%) than patients with uropathies (22.6%) or with other etiologies (5.3%). Patients on dialysis had a higher prevalence of elevated PWV (46.1%) than those under conservative treatment (13.6%). As for dialysis time, there was a higher degree of elevation of PWV among those who received dialysis for one year or more (50%). Among the patients who underwent dialysis for less than one year, the elevation of PWV was not significant (p=0.077). Regarding BP, there was a higher prevalence of elevated PWV (66.7%) among patients classified as stage 2 hypertension than among normotensive patients (13.8%). It is noteworthy that no patient with elevated PWV was observed among those classified as having high BP.

**Table 2:** Clinical and anthropometric characteristics of pediatric patients with chronic kidney disease according to the presence of elevated pulse wave velocity

	Total	Increased PWV	PR	95% CI	p value

		n (%)			
Sex					0.805
Female	22	5 (22.7)	1.00		
Male	35	7 (20.0)	0.88	0.32-2.43	
Age					0.104
6 ⊢12 years old	30	9 (30.0)	1.00	0.11-1.23	
12 ⊢ 18 years old	27	3 (11.1)	0.37		
Etiology					0.016
Glomerulopathy	7	4 (57.1)	1.00		
Uropathy	31	7 (22.6)	0.39	0.16-0.99	0.047
Other	19	1 (5.3)	0.09	0.01-0.69	0.020
Time of disease					0.914
<2 years	12	3 (25.0)	1.00		
2 ⊢ 5 years	15	3 (20.0)	0.80	0.20-3.27	0.756
≥5 years	30	6 (20.0)	0.80	0.24-2.69	>0.999
CKD classification					0.233
Stage 2	10	1 (10.0)	1.00		
Stage 3a	8	1 (12.5)	1.25	0.09-17.02	0.867
Stage 3b	12	1 (8.3)	0.83	0.06-11.70	0.892
Stage 4	10	2 (20.0)	2.00	0.21-18.69	0.543
Stage 5	17	7 (41.2)	4.12	0.59-28.77	0.154
Treatment					0.012
Conservative	44	6 (13.6)	1.00		
Dialysis	13	6 (46.1)	3.38	1.31-8.73	
Time of dialysis					0.031
No dialysis	44	6 (13.6)	1.00		
<1 year	7	3 (42.9)	4.75	0.84-26.71	0.077
≥1 year	6	3 (50.0)	6.33	1.03-38.98	0.047
H/A z-score					0.079
Between -2 and +2	35	4 (11.4)	1.00		
>+2	2	1 (50.0)	4.37	0.83-23.12	0.082
<-2	20	7 (35.0)	3.06	1.02-9.19	0.046
BMI/A z-score					0.661
Between -2 and +2	42	9 (21.4)	1.00		
>+2	6	2 (33.3)	1.55	0.44-5.54	0.496
<-2	9	1 (11.1)	0.52	0.07-3.59	0.506
Blood pressure					0.036
Normotensive	29	4 (13.8)	1.00		
Elevated	5	0 (0.0)			
Stage 1 hypertension	17	4 (23.5)	1.70	0.49-5.95	0.402
Stage 2 hypertension	6	4 (66.7)	4.83	1.65-14.11	0.004

PR: prevalence ratio; 95%CI: 95% confidence interval; PWV: pulse wave velocity; CKD: chronic kidney disease; H/A z-score: score and height for age; BMI/A: body mass index for age.

No significant association between laboratory parameters in altered PWV were found, except for PTH (p=0.044) and LDL-C (p=0.048), *i.e*., no patient with elevated PWV was observed among the 15 patients with PTH within the reference range. However, 12 of the patients with out-of-reference PTH values (≤9 or ≥33) had elevated PWV. Despite the significant association, from a statistical point of view, it was not possible to estimate PR in this case because no patient with altered PWV was observed among those who had PTH values within the reference.

For LDL, there was a higher percentage of patients with altered PWV among those with elevated LDL-C (41.7%) than those presenting normal LDL-C levels (15.9%).

The prevalence of elevated PWV among patients younger than 12 years old was 2.9 times that observed among patients older than 12 years old (95%CI: 1.05-8.40) (Table 3). Patients on dialysis had a prevalence of elevated PWV 4.2-fold higher than that of patients on conservative treatment (95%CI: 1.97-9.13). Patients classified as hypertensive in stage 1 or 2 showed prevalence of elevated PWV a 2.7-fold higher than patients classified as normotensive (95%CI: 1.05-6.95).

**Table 3:** Multivariate analysis of factors associated with elevated pulse wave velocity in pediatric patients with chronic kidney disease

Elevated PWV	adjPR	95%CI	p value
Age 6 ⊢ 12 years old	2.95	1.05-8.40	0.041
Dialysis	4.24	1.97-9.13	<0.001
Stage 1 and 2 hypertension	2.70	1.05-6.95	0.040

adjPR: adjusted prevalence ratio; 95%CI: 95% confidence interval coefficient; PWV: pulse wave velocity.

According to table 4, except for the BP classification, none of the clinical or anthropometric characteristics was associated with elevated cSBP (p>0.05). For the classification of BP, there was a higher percentage of patients with elevated cSBP among those with BP classified as stage 2 (83.3%) than those classified as normotensives (13.8%).

**Table 4:** Clinical and anthropometric characteristics of pediatric patients with chronic kidney disease according to the presence of elevated central systolic blood pressure

	Total	Elevated cSBP	PR	95%CI	p value

		n (%)			
Sex					0.486
Female	22	5 (22.7)	1.00		
Male	35	11 (31.4)	1.38	0.55-3.45	
Age					0.733
6 ⊢12 years old	30	9 (30.0)	1.00		
≥12 years old to 17.5 years old	27	7 (25.9)	0.86	0.37-2.00	
Etiology					0.090
Glomerulopathy	7	3 (42.9)	1.00		
Uropathy	31	11 (35.4)	0.83	0.31-2.20	0.705
Other	19	2 (10.5)	0.25	0.05-1.17	0.079
Time of disease					0.582
<2 years old	12	2 (16.7)	1.00		
2 ⊢ 5 years old	15	4 (26.7)	1.60	0.35-7.30	0.544
≥5 years	30	10 (33.3)	2.00	0.51-7.81	0.319
CKD classification					0.221
Stage 2	10	1 (10.0)	1.00		
Stage 3a	8	4 (50.0)	5.00	0.69-36.37	0.112
Stage 3b	12	2 (16.7)	1.67	0.18-15.80	0.656
Stage 4	10	2 (20.0)	2.00	0.21-18.69	0.543
Stage 5	17	7 (41.2)	4.12	0.59-28.77	0.154
Treatment					0.099
Conservative	44	10 (22.7)	1.00		
Dialysis	13	6 (46.1)	2.03	0.91-4.52	
Time of dialysis					0.269
No dialysis	44	10 (22.7)	1.00		
<1 year	7	3 (42.9)	1.88	0.68-5.20	0.220
≥1 year	6	3 (50.0)	2.20	0.82-5.79	0.110
H/A z-score					0.667
Between -2 and +2	35	9 (25.7)	1.00		
>+2	20	6 (30.0)	1.17	0.49-2.80	0.730
<-2	2	1 (50.0)	1.94	0.44-8.68	0.384
BMI/A z-score					0.497
Between -2 and +2	42	11 (26.2)	1.00		
>+2	9	2 (22.2)	0.85	0.23-3.19	0.808
<-2	6	3 (50.0)	1.91	0.74-4.92	0.181
Blood pressure					0.006
Normotensive	29	4 (13.8)	1.00		
Elevated	5	1 (20.0)	1.45	0.20-10.45	0.712
Stage 1 hypertension	17	6 (35.3)	2.56	0.84-7.80	0.098
Stage 2 hypertension	6	5 (83.3)	6.04	2.27-16.06	<0.001

cSBP: central systolic blood pressure; PR: prevalence ratio; 95% CI: 95% confidence interval coefficient; CKD: chronic kidney disease; H/A z-score: score and height for age; BMI/A: body mass index for age.

There was no significant association between elevated cSBP and laboratory tests (p>0.05). The variables independently associated with abnormal cSBP found through the multivariate model were type of treatment and BP. The prevalence of elevated cSBP among patients on dialysis was approximately 2.1 times that observed among those under conservative treatment (95%CI: 1.07-4.02; p=0.031). Patients classified with stage 1 or 2 of hypertension had a prevalence of elevated cSBP 3.3 times that observed among patients classified as normotensive (95%CI: 1.36-7.94; p=0.008).

## DISCUSSION

This study analyzed PWV and cSBP and their associations with clinical, anthropometric, and laboratory data of children and adolescents at different stages of CKD. Patients under dialysis treatment had a higher prevalence of elevated PWV and elevated cSBP than patients under conservative treatment. Regarding BP, patients on stages 1 and 2 of hypertension had higher prevalence of elevated PWV and cSBP. Children younger than 12 years old had higher prevalence of elevated PWV than those aged 12 years or older, but did not predict higher cSBP.

Cardiovascular disease is the main cause of mortality in patients with CKD, and its manifestation is subclinical in most cases. In recent years, the number of studies describing cardiovascular changes in children and adolescents has increased, and the need to include cardiovascular assessment in the clinical routine of care for patients with CKD has led to the search for rapid assessment methods validated for the pediatric population and independent operators.^(21)^ The analysis of PWV and cSBP is already well established for evaluating arterial stiffness, and the use of the indirect oscillometric method has advanced in the literature.

Although there are different methods for assessing PWV, many have not been standardized for children. Tonometry, which is considered the “gold standard”, is a method that is difficult to apply in pediatrics. Even with the child cooperating during the test, the signal may be not adequately detected in the arteries of young children. The oscillometric methods of PWV analysis, which use pressure cuffs, have the advantage of being rapid, consistent, and operator independent.^(22)^

The first studies describing vascular changes in the pediatric population mainly focused on patients with terminal CKD, especially those on dialysis and post-transplantation. In 2006, Covic et al. analyzed 14 children on hemodialysis and compared them with a control group; they found a higher PWV in hemodialysis patients.^(23)^ This finding was also observed in transplanted patients: Briese et al. analyzed 36 transplanted children and compared them with a Control Group; they also found elevated PWV in the transplant group, and systolic pressure showed significant association with elevated PWV in the multivariate analysis.^(24)^ Kis et al.^(25)^ included 11 patients with a mean age of 14 years old (standard deviation = 4.1 years) who were compared with a control population of 133 healthy children. There was no difference in PWV between patients on dialysis and the normal population when matched by age; however, when matched by height, the PWV of patients on dialysis was significantly higher.^(25)^ This finding indicates that PWV is directly related to body size.

In this study, we used a reference population validated with the device utilized, and PWV was considered elevated when above the 95^th^percentile of the reference population by sex and height. The prevalence of elevated PWV was 21.1%. In a multicenter European study (The 4C Study) which included 12 countries and 688 pediatric patients under conservative treatment, PWV was also evaluated using reference values for height and age. Elevated PWV, defined as above the 95t^h^ percentile, was observed in 20.1% of patients.^(26)^

The interest in studying early cardiovascular changes in pediatric patients with CKD has grown in recent years. The demonstration of PWV changes and their incorporation in the evaluation and long-term follow-up of children and adolescents with terminal CKD may help in the development of strategies to decrease cardiovascular morbidity after the onset of kidney disease. The long-term consequences of vascular injury are particularly important in the pediatric population because these children and adolescents will be under renal replacement therapy for a long time.

In 2016, Taşdemir et al. published a study with 25 pediatric patients with stage 2 CKD and elevated PWV compared to the control group. The elevation in PWV was independently associated with arterial hypertension, which was also found in our study. In another study with 188 children with CKD under conservative treatment (with GFR ranging from 94 to 30mL/min/1.73m^2^),^(7)^ Sinha et al. observed a higher PWV than in the Control Group; however, there was no association of elevated PWV with the stage of the disease, only with hypertension, which suggests that vascular injury begins early.^(27)^

Parathyroid hormone was associated with elevated PWV in the univariate analysis but could not be included in the multivariate analysis because no patient with normal PTH had altered PWV. Parathyroid hormone, which is altered in mineral and bone disorders, is an important nontraditional risk factor in the development of CVD in chronic renal patients.^(28)^

When analyzing the age range of patients and its relationship with PWV, a statistically significant association was found between elevated PWV and younger age (<12 years old). The studies in which older age was associated with elevated PWV^(7,28)^ used the raw value of PWV and did not compare patients by height. The immaturity of the cardiovascular system and the low functional reserve of the young child would explain this higher prevalence of vascular injury in this group. Further studies in infants and preschool children with CKD are necessary.

Central blood pressure is not well studied in pediatrics, but some studies in adults suggest that it is more related to cardiovascular risk, such as carotid intima-media thickness and left ventricular hypertrophy, than peripheral (brachial) pressure.^(29)^ The CAFE study, with 2,199 adult patients, observed that antihypertensive drugs had different effects on central aortic pressure despite having similar effects on peripheral pressure.^(30)^In their 2015 study, Sinha et al., also observed that children with CKD and peripheral BP above the 75^th^ percentile had higher central blood pressure than the Control Group.^(27)^ The measurement of central arterial pressure can help in the diagnosis of hypertension and in the therapeutic management of children, and there are already tables with reference values per height, age, and sex for cSBP, such as the one used in this study.^(11)^

Some limitations of the current study deserve comment. The number of children included could be considered relatively small, but it is comparable to previous studies. The analysis of patients undergoing conservative treatment and dialysis was combined to increase the statistical power. However, in the multivariate analysis, the dialysis variable was independently associated, thus controlling for possible clinical differences in relation to conservative treatment. Finally, as it was a cross-sectional study, the analysis was not done over time, and further prospective studies are needed to confirm our findings in this cross-sectional analysis.

## CONCLUSION

In the studied pediatric patients with chronic kidney disease, the prevalence of elevated pulse wave velocity and elevated central systolic blood pressure is common. Among several associations observed in the univariate analysis, younger age, dialysis, and hypertension were independently associated with elevated pulse wave velocity. Dialysis and hypertension were independently associated with elevated central systolic blood pressure.
